# Supplementary material for: Effect of Oral Supplementation of Healthy Pregnant Sows with Sucrosomial Ferric Pyrophosphate on Maternal Iron Status and Hepatic Iron Stores in Newborn Piglets
Source: Animals (Basel). 2020 Jun 29;10(7):1113. doi: 10.3390/ani10071113 (PMC7401508; doi:10.3390/ani10071113)
Supplement: Supplementary file 1 [file animals-10-01113-s001.pdf]

Supplementary Table 1. The composition and chemical analysis of the basal diet of pregnant sows. (g/kg as-fed basis if not stated otherwise)

**Ingredients (g / kg)**		**Chemicals composition (g / kg)**	
Ground barley	400.0	ME(MJ/kg)	13.7
Ground wheat	163.0	Dry matter	887.0
Ground maize	250.0	Crude protein	132.0
HP300[a]	100.0	Crude fat	71.7
Premix[b]	65,1	NDF	120.9
Calcium formate	15.2	ADF	40.3
Probiotic[c]	0.7	Ash	45.0
NaCl	6.0	Phosphorus	6.2
		Calcium	8.3
Total	1000g	Fe(mg/kg)	40.0
		Cu(mg/kg)	3.0
		Zn(mg/kg)	15.0
		Mn(mg/kg)	30.0
		Lysine	9.9
		Methionine	2.9
		Threonine	6.8
		Tryptophan	1.8

Supplementary Table 1. [a] Soy protein concentrate. [b] Premix provided the following per kg of diets: Na 0.28 g, Ca 2.15 g, P 0.7 g; Mg 0.1 g; Lysine 0.2 g; Fe 37 mg: Mn 30 mg; I 0.75 mg; Zn 15 mg; Cu 2.55 mg; Co 0.1 mg; Se 0.1 mg, Methionine 50 mg; Threonine 90 mg, Tryptophan 15 mg, Histidine 17 mg, Vitamin A 10000 IU; Vitamin D3 1000 IU; Vitamin E 20 mg; Vitamin K3 1.5 mg; Vitamin B1 0.3 mg; Vitamin B2 0.8 mg; Vitamin B6 1.0 mg; Vitamin B12 10 µg; Niacin 5 mg; Folic acid 0.5 mg; Biotin 20 µg; Nicotinic acid 6.5 mg; Pantothenic acid 8 mg; Choline 25 mg. [c] Bonvital, lactic acid bacteria - Enterococcus faecium (DSM 7134, 4b1841), 1 x 10^{10} CFU/g,(SCHAUMANN Agri International GmbH).

Supplementary Table 2. Antibodies used in immunofluorescence analyses.

**Target protein**	**Primary Ab**	**Primary Ab dilution**	**Secondary Ab**	**Secondary Ab with fluorochrome**	**Secondary Ab with fluorochrome dilution**
TfR1	Transferrin receptor monoclonal antibody Mouse monoclonal, Thermo Fisher #136800	1:200	Goat anti-mouse polyclonal, #A5278 (Sigma-Aldrich)	Goat anti-Mouse IgG (H+L), Alexa Fluor 594 A-11005 **Invitrogen**	1:2000

Fpn	Rabbit Anti-Mouse Metal Transporter Protein1/Ferroportin (MTP1/IREG1/Fpn) IgG aff. pure<br>#MTP11-A<br>Alpha Diagnostic Intl. Inc	1:200	Goat anti-rabbit polyclonal, #A6154 (Sigma-Aldrich)	Goat anti-Rabbit IgG (H+L) Alexa Fluor Plus 488 A32731<br>**Invitrogen**	1:2000
DMT1	Anti-Human Natural Resistance-Associated Protein 2 (DMT1/NRAMP2, with IRE)<br>#NRAMP22-A<br>Alpha Diagnostic Intl. Inc	1:200	Goat anti-rabbit polyclonal, #A6154 (Sigma-Aldrich)	Goat anti-Rabbit IgG (H+L), Alexa Fluor Plus 488 A32731<br>**Invitrogen**	1:2000

Supplementary Table 3. Gene specific primers sequences used in RT-PCR analyses.

**Target gene**	**Forward primer (5′→3′)**	**Reverse primer (5′→3′)**	**Accession number**
Actb	CAACTTCTGCAACGAGCGCTT	GCGCCCCCGAGCCTTGATCTC	AY550069.1
TfR1	AGCCTTACTGTATGCCACGT	ATTATGCCCCTCGTGAAGCT	NM_214001.1
Fpn	TCGCCTAGTGTCATGACCAG	CAGAAACACAGACACCGCAA	NM_001128440.1
DMT1	GCAGGTGGTTGACGTCTGTA	CACGCCCCCTTTGTAGATGT	NM_001128440.1
HPRT	GGCCATCACATCGTAGCCCT	TCGCCCGTTGACTGGTCATT	NM_001032376.2
BMP6	TTGTGAACCTGGTGGAGTACG	GATTCGGAATTCTGCAGCCG	NM_001168001.1

Suplementary Figure 1.

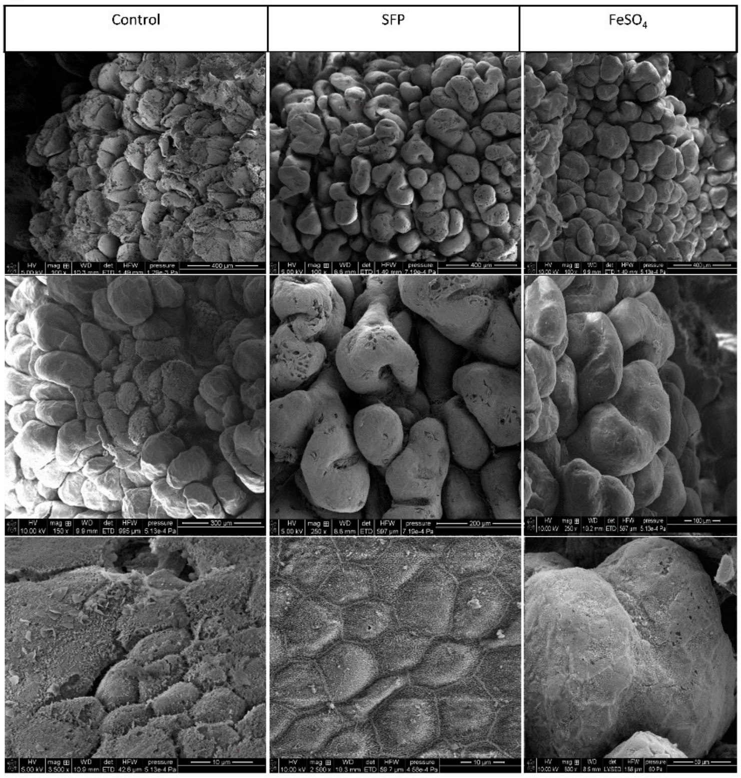

Supplementary Figure 1. Illustration of the surface of placental tissues acquired using T.E.M microscopy.Used magnifications range from 100x, to 3500x.

Suplementarny Figure 2.

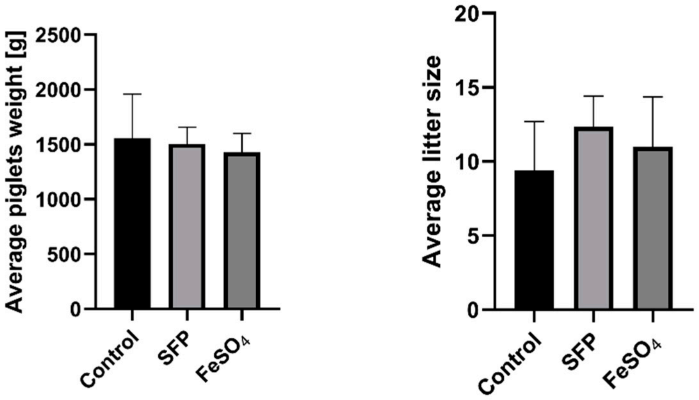

Supplementary Figure 2. Pregnancy outcome. One day piglets weight recorded at delivery and presented as mean and S.D for every experimental group in grams, Litter size recorded at delivery, presented as mean and S.D for every experimental group.

SPF – SiderAL® Forte formulation:

INGREDIENTS: - Sucrosomial iron® (iron pyrophosphate – 6 mg of Fe/g of powder, pregelatinized rice starch, saccharic esters of fatty acids, sunflower lecithin supported on glucose syrup, milk proteins, tricalcium phosphate)
